# Characterization of atherosclerotic carotid plaque using MATCH: initial clinical experience

**DOI:** 10.1186/1532-429X-17-S1-O72

**Published:** 2015-02-03

**Authors:** Wei Yu, Zhaoyang Fan, Lixin Yang, Yibin Xie, Li Dong, Zhanhong Wang, Yanni Du, Xiaoming Bi, Jing An, Tianjing Zhang, Gerhard Laub, Zhaoqi Zhang, Debiao Li

**Affiliations:** 1 Department of Radiology, Beijing anzhen Hospital, Capital Medical University, Beijing, China; 2 Biomedical Imaging Research Institute, Cedars-Sinai Medical Center, Los Angeles, CA, USA; 3 Department of Bioengineering, University of California, Los Angeles, CA, USA; 4 MR R&D, Siemens Healthcare, Los Angeles, CA, USA; 5 MR Collaborations NE Asia, Siemens Healthcare, Beijing, China

## Background

Characterization of carotid artery morphology and plaque composition requires multiple measurements with different contrast-weightings, which are limited by long scan time, image misregistration, and expertise-dependency in image interpretation. Recently, a new method,3D Multi-contrast ATherosclerosis CHaracterization (MATCH) has been developed to overcome the above drawbacks by acquiring multi-contrast weighted images in one scan [1] This study was conducted to determine the accuracy of MATCH in the characterization of plaque morphology and composition in patients.

## Methods

Thirty patients scheduled for carotid revascularization underwent preoperative carotid MRI with MATCH and the conventional multi-contrast protocol (T1W, T2W,TOF,CE-T1W) in the same examination with a 3T scanner(Table 1) and 8-channel carotid coil. All image sets were processed using plaque analysis software (MRI-Plaque View, VPDiagnostics). Blinded image review for anatomy and composition identification was performed by 2 radiologists (with 2 and 9-year experience in carotid plaque MR characterization). Images from each artery underwent location matching process (including image reformation in 3D TOF) to account for inconsistency in slice number and thickness between the two protocols and inter-scan motion. Quantitative area measurements of the lumen and wall of the bilateral carotid arteries were obtained from T2-w images. The normalized wall index (NWI) was calculated by dividing the wall area by the total vessel area (lumen+wall). The presence of intraplaque hemorrhage (IPH), calcification (CA), and lipid-rich necrotic core (LRNC) were determined using the criteria for the MATCH protocol [1] and those in a review article for the conventional protocol [2] The paired t-test was used to compare the two measurements. Cohen kappa (K) was computed to quantify the agreement in the detection of components between the two protocols.

**Table 1:** Scan Parameters of Conventional and MATCH Protocol

	TOF	T1W /TSE	T2W TSE	CE-T1W	MATCH
TE/TR (ms)	3.6/21	12.0/800	59.0/4000	12.0/800	4.2/10.8

In-plane resolution (mm2)	0.87×0.52	0.55/0.63× 0.55/0.63	0.55/0.63× 0.55/0.63	0.55/0.63× 0.55/0.63	0.55/0.63× 0.55/0.63

Number of slices	84	18	16	16	16

Slice thickness (mm)	1	2	2	2	2

Number of Average	1	2	2	2	2

Flip angle	25	136-170	120-160	136-170	8

Echo train length	-	7	12	7	55-67

Bandwidth (MHz)	250	407	407	407	130

Scan time (min:sec)	2:27	2:56	3:24	2:56	4:45

## Results

The two protocols were successfully performed in all 30 patients. The scan time was about 15 min for the conventional protocol and 5 min for the MATCH protocol.The 30 patients bilateral artery yielded 898 matched MRI cross-sectional slices for analysis. The statistical analysis shown no significant difference of mean area of lumen (36.27 vs 35.72 mm^2^, p=0.62), wall (42.80 vs 43.64 mm^2^, p=0.82) and NWI (59.21 vs 59.81, p=0.72) measured by MATCH and conventional protocols. Moderate to good agreement was seen between 2 protocols in the detection of plaque components (LR/NC k=0.84, CA k=0.802, IH k=0.773).

## Conclusions

Our preliminary clinical study suggests that MATCH has similar performance in the evaluation of carotid plaque to the conventional multi-contrast protocol. Shorter scan time, less image misregistration and relatively simplified criteria for component have substantially reduced the examination failure rate and shorten the time consuming of interpretation.MATCH is a promising CMR imaging method for assessing the risk of plaque disruption in a clinical workup.

## Funding

Beijing health system of high-level technical personnel training project 2013-2-005.

